# Afecções Pericárdicas em Pacientes com COVID-19: Uma Possível Causa de Deterioração Hemodinâmica

**DOI:** 10.36660/abc.20200474

**Published:** 2020-09-18

**Authors:** Fábio Fernandes, Felix José Alvarez Ramires, Fábio Danziato Fernandes, Marcus Vinicius Simões, Evandro Tinoco Mesquita, Charles Mady

**Affiliations:** 1 Universidade de São Paulo Faculdade de Medicina Hospital das Clínicas São Paulo SP Brasil Universidade de São Paulo Faculdade de Medicina Hospital das Clínicas Instituto do Coração,São Paulo, SP - Brasil; 2 Centro Universitário São Camilo São Paulo SP Brasil Centro Universitário São Camilo,São Paulo, SP - Brasil; 3 Universidade de São Paulo Faculdade de Medicina de Ribeirão Preto Ribeirão Preto SP Brasil Universidade de São Paulo Faculdade de Medicina de Ribeirão Preto,Ribeirão Preto, SP - Brasil; 4 Universidade Federal Fluminense Niterói RJ Brasil Universidade Federal Fluminense,Niterói, RJ - Brasil

**Keywords:** Coronavirus-19, COVID-19, Betacoronavirus/complicações, Pandemia, SARS-CoV-2, Hipóxia, Complicações Cardiovasculares, Inflamação Sistêmica, Trombose

## Introdução

O curso clínico da infecção por SARS-CoV-2 é caracterizado por sintomas do trato respiratório, febre, tosse, dor de garganta, fadiga e complicações relacionadas à síndrome do desconforto respiratório agudo. No entanto, alguns pacientes que inicialmente apresentam sintomas leves poderão subsequentemente apresentar deterioração clínica, a qual ocorre aproximadamente uma semana após o início dos sintomas.^1^

A progressão aguda da COVID-19 pode ser dividida em três fases distintas: infecção precoce, fase pulmonar e hiperinflamação. No entanto, pode haver sobreposição significativa entre as diferentes fases em qualquer paciente.^2^

O envolvimento cardíaco é uma característica proeminente da COVID-19 e está associado a pior prognóstico. Os mecanismos de lesão cardíaca são múltiplos, sendo descritos aumento do estresse cardíaco secundário a insuficiência respiratória, hipoxemia, infecção miocárdica direta pelo SARS-CoV-2, doença microvascular ou microtrombose e injúria indireta secundária a inflamação sistêmica.^3^

Frente a um paciente com COVID-19 que apresenta piora clínica, devemos incluir como possíveis diagnósticos diferenciais: pneumonia em evolução pelo SARS-CoV-2, exacerbação aguda da insuficiência cardíaca crônica, síndrome coronariana aguda, embolia pulmonar aguda, miocardite e afecções pericárdicas.^4^

Diante do exposto, realizou-se uma revisão não sistemática da literatura a fim de abordar os principais estudos que sugerem comprometimento pericárdico em pacientes com COVID-19. A base de dados consultada foi a PubMed (www.ncbi.nlm.nih.gov/pubmed). Foram selecionados artigos originais e de revisão realizados em humanos, redigidos em português e inglês. Os seguintes descritores MeSH foram utilizados como palavras-chave: COVID-19, *cardiac tamponade*, SARS-CoV-2 e *pericarditis*.

Neste trabalho, objetivamos revisar as evidências disponíveis até o momento em relação ao envolvimento pericárdico de pacientes com SARS-CoV-2, suas formas de apresentação clínica, mecanismos fisiopatológicos e possibilidades terapêuticas, salientando a possiblidade de comprometimento hemodinâmico pela ocorrência de tamponamento cardíaco (Quadro 1).

**Quadro 1:** – Reconhecimento de pericardiopatias em pacientes com COVID-19

**1. Paciente com COVID-19 e suspeita de pericardite:** • dispneia, taquipneia, edema de membros inferiores; • taquicardia; • hipotensão; • pulso paradoxal.
**2. Exames complementares:** • ECG (baixa voltagem); • troponina e CKMB (miopericardite); • ecocardiograma transtorácico (sinais de tamponamento).
**3. Diagnóstico diferencial:** • síndrome coronariana aguda; • cardiomiopatia relacionada a sepse (tempestade de citocinas); • miocardite; • síndrome de Takotsubo.
**4. Investigações adicionais:** • ressonância magnética cardíaca, se possível; • angiotomografia de coronárias.
**5. Considerar periocardiocentese**

## Afecções Pericárdicas

O derrame pericárdico é a apresentação clínica mais comum das pericardiopatias. Processos infecciosos causados por agentes virais, bacterianos ou fúngicos podem cursar ou não com o acometimento do pericárdio. Em caso de pericardite, encontraremos, em sua forma aguda, manifestações clínicas como dor torácica tipo pleurítica e atrito pericárdico, além de alterações no traçado eletrocardiográfico. A condição mais comumente associada ao derrame pericárdico é a pericardite aguda de etiologia viral, frequentemente uma condição autolimitada.^5^

Nos casos de pacientes com COVID-19, a ocorrência de derrame pleural, linfadenopatia, cavitação, sinal de halo da tomografia de tórax, pneumotórax e derrame pericárdico é incomum, mas pode ser observada com a progressão da doença.^6^ A ação do vírus no pericárdio pode ser por efeito citotóxico direto e/ou por mecanismo imunomediado.^7^

O primeiro relato de tamponamento cardíaco secundário à infecção pela COVID-19 foi feito em 30 março de 2020, em uma paciente de 47 anos (Quadro 2). A paciente referia sintomas de febre e tosse seca e não apresentava comorbidades cardiovasculares, como hipertensão ou diabetes, mas apresentava antecedente de miopericardite e teste positivo para COVID-19 em *swab* nasofaríngeo. Evoluiu com instabilidade hemodinâmica, sendo diagnosticado tamponamento cardíaco e identificada necessidade de pericardiocentese – na qual 540 mL de líquido serossanguinolento foram retirados. Esse caso chamou atenção da comunidade médica para a possibilidade do comprometimento pericárdico evoluir para tamponamento cardíaco e ser um fator responsável pela deterioração hemodinâmica em pacientes com COVID-19.^8^

**Quadro 2:** – Casos de afecções pericárdicas e COVID-19

Data publicação ^(ref.)^ País	Idade (anos)	Sexo	Antecedentes CV	Tamponamento/Pericardiocentese	Sintomas prévios COVID-19	Tratamento coadjuvante
30/03/2020 Reino Unido^8^	47	F	Miopericardite	Sim / Sim	Sim	Não relatado
27/03/2020 Itália^9^	53	F	Sem antecedentes	Não / Não (miopericardite)	Sim	Lopinavir/Ritonavir Corticoide/hidroxicloroquina
23/04/2020 Itália^10^	59	M	Cardiopatia isquêmica aguda (revascularização miocárdica)	Sim / Sim	Sim	Lopinavir/Ritonavir Enoxaparina/hidroxicloroquina
23/04/2020 Estados Unidos^7^	67	F	Miocardiopatia isquêmica FE 40%	Sim / Sim	Sim	Colchicina, hidroxicloroquina, corticoide
12/05/2020^11^ Arábia Saudita	41	F	Sem antecedentes	Sim / Sim	Sim	Azitromicina e Hidroxicloroquina
06/05/2020^12^ Estados Unidos	70	F	Cardiopatia isquêmica, hipertensão e diabetes	Sim / Sim	Sim	Colchicina

Outro caso de tamponamento pericárdico foi identificado em uma paciente de 67 anos com antecedente de miocardiopatia isquêmica e fração de ejeção de 40% que, após uma semana do início de sintomas respiratórios, desenvolveu fadiga, hipotensão e taquicardia. Além disso, o eletrocardiograma evidenciou baixa voltagem, com os marcadores séricos troponina e BNP normais. Devido à persistência dos sintomas, realizou-se ecocardiograma, o qual evidenciou tamponamento pericárdico. Em seguida, foi realizada drenagem de 800 mL de líquido sero-hemorrágico com características de exsudato. Logo após a pericardiocentese, a paciente evoluiu para cardiomiopatia induzida por estresse, caraterizada pelo abaulamento apical transitório (síndrome de Takotsubo), conforme achados clínicos, laboratoriais, eletro e ecocardiográficos.^7^

Uma paciente de 53 anos, sem antecedentes mórbidos, procurou serviço uma semana após o início dos sintomas. Deu entrada com hipotensão e alterações eletrocardiográficas com supradesnivelamento difuso do segmento ST e elevação de biomarcadores, como troponina e NT-proBNP, mas com coronárias angiograficamente normais. Foi solicitado exame PCR de *swab* de nasofaringe, o qual testou positivo para SARS-CoV-2. A ressonância magnética cardíaca evidenciou edema e realce tardio miocárdicos, além de derrame pericárdico circunferencial compatível com miopericardite.^9^

Um paciente de 59 anos deu entrada com síndrome coronariana aguda e foi submetido a cateterismo cardíaco, o qual evidenciou lesões triarteriais. O paciente recebeu tratamento de revascularização miocárdica. No 22º pós-operatório, iniciou sintomas de febre e dispneia e apresentou PCR positivo para SARS-CoV-2, com tomografia de tórax compatível com pneumonia por SARS-CoV-2.^10^ No 23º dia de infecção, o paciente evoluiu com dor precordial, dispneia, hipotensão e taquicardia. O ecocardiograma evidenciou derrame pericárdico circunferencial com sinais de tamponamento de câmaras direitas, sendo drenados 250 mL de líquido serossanguinolento.

Em outro caso, uma paciente de 70 anos com antecedentes de doença arterial coronária aguda (infarto do miocárdio sem supradesnivelamento do segmento ST duas semanas antes da internação, com angioplastia recente) deu entrada com sintomas respiratórios. No segundo dia de internação, apresentou dor torácica sugestiva de pericardite aguda, evoluindo com tamponamento cardíaco, intubação orotraqueal e instabilidade hemodinâmica com necessidade de drogas vasoativas. Houve melhora clínica e hemodinâmica apenas após a pericardiocentese.^11^

Outro caso de COVID-19 e tamponamento cardíaco envolveu uma paciente de 41 anos, sem antecedentes prévios, que havia procurado o serviço com sintomas respiratórios 10 dias antes da internação. Realizou-se pericardiocentese e evidenciou-se exsudato no líquido pericárdico, com elevados níveis de DHL e albumina.^12^

Nesses seis primeiros casos, observamos que pacientes com SARS-CoV-2 podem apresentar comprometimento pericárdico na evolução clínica. O período de diagnóstico e piora clínica, em geral após a primeira semana da infecção, coincide com a fase de aumento de citocinas inflamatórias, provavelmente com mecanismos autoimunes envolvidos no mecanismo etiopatogênico do derrame.

Alguns autores acreditam que, em pacientes com miocardite secundária ao coronavírus, a infecção viral pode ser o fator inicial, desencadeando injúria imunomediada. Em estudos de necropsia, observa-se infiltrado inflamatório mononuclear nos tecidos cardíacos, porém sem presença de inclusão viral em alguns casos.^13^ A rápida recuperação da função cardíaca e o leve aumento na carga viral sugerem que, além da replicação viral no miocárdio, é possível que a resposta imune, uma tempestade de citocinas, realmente possa desempenhar um papel significativo na fisiopatologia da agressão.^14^

A liberação excessiva de citocinas pode ser observada em grande variedade de doenças sistêmicas, incluindo doenças infecciosas, reumáticas e neoplásicas, as quais podem levar ao comprometimento pericárdico.^15^

Imunologistas e patologistas observaram infiltrado de macrófagos na necropsia, podendo indicar que a viremia e imunidade inata dominam o quadro clínico antes que o infiltrado linfocitário possa ocorrer. Observam-se parâmetros inflamatórios aumentados, entre eles a proteína C-reativa e as citocinas pró-inflamatórias (IL-6, TNFα, IL-8l).^15^

Dessa forma, um estado hiperinflamatório induzido pela ação de citocinas pode levar à insuficiência de múltiplos órgãos e poderia ser responsável pelo comprometimento miocárdico e pericárdico na doença; no entanto, não podemos excluir a possibilidade de parte desse comprometimento ser causado pelo vírus.^16^

O caso do paciente relatado no estudo italiano exibiu um período mais tardio até o aparecimento de pericardite, e a análise química e citológica do líquido pericárdico demonstrou presença de infiltrado inflamatório com predomínio linfocitário e presença de SARS-CoV-2, sugerindo possível efeito direto do vírus nas manifestações cardiológicas. É interessante notar que o paciente apresentava PCR negativo no sangue e na região nasofaríngea, sendo apenas positivo no líquido pericárdico, o que pode sugerir possível reservatório do vírus no pericárdio.^10^

Em cinco dos casos, a apresentação foi de tamponamento cardíaco, uma causa de descompensação clínica de alto risco, mas potencialmente reversível. A decisão de adotar uma técnica invasiva de tratamento é influenciada pelo quadro clínico do paciente. Quando o quadro for evidente, com presença de hipotensão arterial, dispneia e pulso paradoxal, não existe controvérsia quanto ao procedimento. A conduta frente ao derrame pericárdico deve estar embasada no contexto clínico (piora dos sintomas), hemodinâmico (presença ou não de tamponamento) ou pesquisa da etiologia (infecção, tuberculose ou neoplasia).^5^

## Tratamento Farmacológico

A definição do melhor tratamento farmacológico para afecção pericárdica em pacientes com SARS-CoV-2 merece discussão. Os anti-inflamatórios não hormonais (AINHs) estão indicados como primeira opção em todos os casos de pericardite aguda e recorrente, desde que não apresentem contraindicações. Recomenda-se ácido acetilsalicílico (AAS) 800 mg, a cada oito horas, ou ibuprofeno 600 mg, a cada oito horas, em associação com a colchicina.^5^

Um estudo retrospectivo realizado na França^17^ demonstrou que o uso de AINHs para efeito de sintomas prévio à hospitalização por pneumonia desenvolveu formas mais graves da doença e período maior de internação dos pacientes. Por outro lado, o uso de ibuprofeno e acetaminofeno também esteve associado a maior risco de complicações em crianças, principalmente em altas doses cumulativas. Especula-se que o alívio sintomático da dor e febre possa acarretar retardo na introdução precoce de antibióticos.^18^ Os possíveis vieses de que pacientes com infecções virais mais severas, como *influenza* e COVID-19, são mais propensos a utilizar anti-inflamatórios e ibuporofeno poderiam ser fatores confundidores do risco aumentado.

A controvérsia relacionada à segurança do uso de ibuprofeno em pacientes com COVID-19 originou-se na França, quando um infectologista relatou piora dos sintomas em quatro pacientes que haviam feito uso da medicação. O relato foi logo respaldado pelo Ministério da Saúde Francês.^19,20^Por outro lado, há dados que mostram que o ibuprofeno aumenta a expressão de receptores ACE_2._ No entanto, sabemos que evidências de efeitos mecanísticos nem sempre são confirmados por estudos clínicos.^20^Atualmente, as recomendações da Organização Mundial da Saúde e da *Food and Drug Administration* (FDA) não se opõem ao uso de ibuprofeno em casos sintomáticos de COVID-19.^21^ Em resumo, as evidências epidemiológicas recentes não são suficientes para afirmar um elo causal entre efeito deletério e uso de ibuprofeno em pacientes com COVID-19. Dessa forma, deve-se ponderar a relação risco-benefício na decisão sobre o uso desse medicamento em pacientes com COVID-19.^22^

Nos últimos anos, a colchicina vem se destacando como medicação coadjuvante aos AINH tanto no tratamento da pericardite aguda quanto no da recorrente, com excelentes resultados, devendo também ser considerada no tratamento da pericardite em pacientes com SARS-CoV-2. Os efeitos anti-inflamatórios da colchicina estão relacionados à ruptura da função dos microtúbulos, cujo resultado é a inibição dos neutrófilos e moléculas de adesão que interferem no início e na amplificação da inflamação.^5^ Com relação ao uso da colchicina em pacientes com COVID-19, existem pelo menos oito estudos incluídos no *site* https://clinicaltrials.gov/ com o objetivo de testar o efeito do medicamento em atenuar a inflamação sistêmica e/ou miocárdica. Dois dos estudos já estão em andamento: *Colchicine Coronavirus SARS-CoV2 Trial* (COLCORONA) e *The ECLA PHRI COLCOVID Trial*. *Effects of Colchicine on Moderate/High-risk Hospitalized COVID-19 Patients*.

Os corticosteroides são muito utilizados no tratamento da pericardite aguda e de suas recorrências porque melhoram os sintomas e causam diminuição de marcadores inflamatórios. Porém, seu uso é limitado aos casos de intolerância, contraindicações ou falha do tratamento com AINHs e colchicina devido ao aumento de recorrências, como demonstrado no estudo COPE.^5^ Há também indicação para o uso de corticosteroides em casos de etiologias específicas, como doenças autoimunes, porém nas menores doses possíveis. Nessas situações, a diminuição da dose deve ser bem lenta e iniciar apenas após o desaparecimento dos sintomas e a normalização da proteína C-reativa. Doses de 0,25 a 0,5 mg/kg/dia devem ser utilizadas nos casos de pericardite.^5^

De maneira geral, para as afecções pericárdicas, o uso de corticoides em detrimento do uso de AINHs e colchicina é sempre evitado. Contudo, a COVID-19 tem se mostrado uma doença peculiar com características fisiopatológicas distintas, na qual há um estado de hiperinflamação e tempestade de citocinas. Nesses casos, a introdução de corticoides talvez possa ser considerada como primeira opção no tratamento das afecções pericárdicas em pacientes com COVID-19. Para essa doença, fármacos que reduzem a inflamação antes da disfunção de múltiplos-sistemas têm sido preconizados - dessa forma, o uso de corticoides poderia ser justificado. Porém, permanecem dúvidas referentes à dose e ao tempo de tratamento ideais.^16^

Diretrizes internacionais sobre o manejo e tratamento da sepse e do choque séptico recomendam que corticoides sejam utilizados em doses pequenas, por curto período de tempo, e nos casos em que a reposição volêmica e as drogas vasopressoras não estabilizarem o paciente.^23^ Em um estudo de Chen et al.,^24^ 19% dos pacientes com pneumonia receberam corticoides - metilprednisolona, 1-2 mg/kg por dia, e dexametasona, por 3 a 15 dias (mediana de 5 dias). Os potenciais riscos do tratamento com corticoides seriam o retardo na eliminação viral e a ocorrência de infecções secundárias.^15^

O SARS-CoV-2 foi inicialmente identificado no lavado pulmonar coletado de um paciente com pneumonia, no final de dezembro de 2019. Desde então, tem-se isolado o vírus no trato respiratório, nas fezes, na conjuntiva e no sangue. Recentemente, tivemos relato da presença do vírus no líquido pericárdico de um paciente com tamponamento cardíaco submetido a pericardiocentese.^10^ Dessa forma, o uso de corticoides seria especulativo nessa situação.

Observamos que alguns pacientes com afecção pericárdica e COVID-19 utilizaram hidroxicloroquina como parte do tratamento da pericardite. No entanto, não há evidência da eficácia e segurança desse medicamento nos pacientes. Pacientes com COVID-19 apresentam quadro de inflamação sistêmica e muitos desenvolvem miocardite concomitante; tal quadro inflamatório pode levar a arritmias ventriculares graves, e a introdução de hidroxicloroquina, um medicamento que aumenta o intervalo QT, poderia potencializar a ocorrência de *torsade de points.*

O tocilizumab, um anticorpo monoclonal antirreceptor de IL-6, está sendo testado em estudos multicêntricos randomizados em pacientes com COVID-19 que apresentam aumento de IL-6 e que possam se beneficiar do tratamento em casos de síndrome de tempestade de citocinas, reduzindo a inflamação miocárdica.

Outra característica clínica proeminente em pacientes com as formas graves de COVID-19 são as manifestações de lesão endotelial e trombose microvascular, podendo mimetizar vasculite, inclusive com casos de amputação de extremidades. No estudo patológico, observa-se infiltração de monócitos e linfócitos ao redor dos vasos sanguíneos com hiperplasia e espessamento da parede do vaso, além de trombos em microvasos; altos níveis de anticorpos antifosfolípides, anticardiolipina e anti-β_2_ glicoproteína estiveram associados a trombose.^15^ Dessa forma, alguns estudos recomendam o uso de anticoagulantes^25^ para tentar evitar fenômenos tromboembólicos e, como observado nos casos relatados anteriormente, os clínicos devem estar atentos, nessa situação, para o risco aumentado de ocorrência de hemopericárdio e tamponamento.

Recentemente, foi publicado um registro internacional de pacientes com casos suspeitos e confirmados de COVID-19 em 69 países de seis continentes, totalizando 1.216 casos. Os pacientes foram submetidos a ecocardiograma, e observou-se que a maioria dos casos apresentava anormalidades não específicas de disfunção ventricular. Entretanto, observaram-se novo infarto do miocárdio, miocardite e cardiomiopatia de Takotsubo em uma minoria dos casos. Em 11 casos (1%), evidenciou-se presença de tamponamento pericárdico, o que demonstra a importância do tema e a possibilidade da utilização de métodos complementares, como o ecocardiograma, para identificar e modificar diretamente a conduta clínica e, consequentemente, o tratamento desses pacientes.^26^

A cardiovigilância dos pacientes que desenvolveram comprometimento pericárdico é de fundamental importância, com o objetivo de avaliar recorrências e/ou evolução para formas constritivas a longo prazo.
