# Supplementary material for: Afecções Pericárdicas em Pacientes com COVID-19: Uma Possível Causa de Deterioração Hemodinâmica
Source: Arq Bras Cardiol. 2020 Sep 18;115(3):569–73. [Article in Portuguese] doi: 10.36660/abc.20200474 (PMC9363091; doi:10.36660/abc.20200474)
Supplement: Translation

# Translation: Pericardial Affections in Patients with COVID-19: A Possible Cause of Hemodynamic Deterioration

**Authors:** Fábio Fernandes, Felix José Alvarez Ramires, Fábio Danziato Fernandes, Marcus Vinicius Simões, Evandro Tinoco Mesquita, Charles Mady

**Affiliations:** 1 Brazil Universidade de São Paulo Faculdade de Medicina Hospital das Clínicas Instituto do Coração, São Paulo, SP - Brazil; 2 Brazil Centro Universitário São Camilo, São Paulo, SP - Brazil; 3 Brazil Universidade de São Paulo Faculdade de Medicina de Ribeirão Preto, Ribeirão Preto, SP - Brazil; 4 Brazil Universidade Federal Fluminense, Niterói, RJ - Brazil

**Keywords:** Keywords, Coronavirus-19, COVID-19, Betacoronavirus/complications, Pandemics, SARS-CoV-2, Hypoxia, Cardiovascular Complications, Systemic, Inflammation, Thrombosis

## Introduction

The clinical course of the SARS-CoV-2 infection is characterized by respiratory symptoms, fever, cough, sore throat, fatigue, and complications related to the acute respiratory distress syndrome. However, some patients with initially mild symptoms can present clinical deterioration approximately one week after onset of the symptoms.^1^

The acute progression of COVID-19 can be divided into 3 different phases: early infection, pulmonary phase, and hyperinflammation, and significant overlaps between these phases can occur.^2^

Cardiac impairment is a prominent characteristic of COVID-19 and is associated with poor prognosis. Its mechanisms include increased cardiac stress caused by respiratory failure, hypoxemia, direct myocardial infection by SARS-CoV-2, microvascular thrombosis, and secondary injury due to systemic inflammation.^3^

When treating a patient with COVID-19 and clinical worsening, possible differential diagnoses should be considered: ongoing pneumonia caused by SARS-CoV-2, acute exacerbation of chronic heart failure, acute coronary syndrome, acute pulmonary embolism, myocarditis, and pericardial affections.^4^

Considering these findings, we performed a non-systematic review of the literature in search of the main studies suggesting pericardial impairment in patients with COVID-19. The examined database was PubMed (www.ncbi.nlm.nih.gov/pubmed). We selected original articles and reviews involving human participants, written in Portuguese or English, using the following medical subject headings (MeSH): cardiac tamponade, SARS-CoV-2, and pericarditis.

We aimed to review the current evidence on pericardial impairment in patients with COVID-19, along with forms of clinical presentation, pathophysiological mechanisms, and therapeutic possibilities, and to highlight the risk of hemodynamic impairment due to cardiac tamponade (Table 1).

**Table 1:** – Recognizing pericardial diseases in patients with COVID-19

**1. Patient with COVID-19 and suspected pericarditis:** • dyspnea, tachypnea, lower limb edema; • tachycardia; • hypotension; • paradoxical pulse.
**2. Complementary examinations:** • electrocardiogram (low voltage); • troponin and CK-MB (myopericarditis); • transthoracic echocardiogram (signs of tamponade).
**3. Differential diagnosis:** • acute coronary syndrome; • sepsis-induced cardiomyopathy (cytokine storm); • myocarditis; • Takotsubo cardiomyopathy.
**4. Additional investigations:** • cardiovascular magnetic resonance imaging, if available; • coronary computed tomography angiography.
**5. Consider pericardiocentesis**

## Pericardial Affections

Pericardial effusion is the most common clinical presentation of pericardial diseases. Infections caused by viral, bacterial, or fungal agents can occur with or without pericardial affection. The acute phase of pericarditis presents clinical characteristics such as pleuritic chest pain and pericardial friction rub, in addition to electrocardiographic abnormalities. The condition most commonly associated with pericardial effusion is viral acute pericarditis, which is frequently a self-limited condition.^5^

In patients with COVID-19, the occurrence of pleural effusion, lymphadenopathy, cardiovascular cavitation, the halo sign in chest imaging, pneumothorax, and pericardial effusion is uncommon, but has been observed throughout the disease progression.^6^ Viral action in the pericardium can occur through a direct cytotoxic effect and/or an immune-mediated mechanism.^7^

The first report of cardiac tamponade secondary to COVID-19 was on March 30, 2020, in a 47-year-old female patient (Table 2). The patient presented with fever and dry cough, tested positive for COVID-19 in a nasopharyngeal swab test, and did not have cardiovascular comorbidities such as hypertension or diabetes; however, there was a history of myopericarditis. The patient developed hemodynamic instability and cardiac tamponade and required pericardiocentesis, which drained 540 mL of serosanguineous fluid. This case attracted the attention of the medical community to the possibility of pericardial impairment evolving into cardiac tamponade and being partly responsible for the hemodynamic deterioration seen in patients with COVID-19.^8^

**Table 2:** – Cases of COVID-19 with pericardial impairment

Date of publication^(ref.)^ Country	Age (years)	Sex	Cardiovascular history	Tamponade/ pericardiocentesis	Previous COVID-19 symptoms	Ancillary treatment
March 30, 2020^8^ United Kingdom	47	F	Myopericarditis	Yes/Yes	Yes	Not reported
March 27, 2020^9^ Italy	53	F	No history	No/No (myopericarditis)	Yes	Lopinavir/ritonavir corticosteroid/ hydroxychloroquine
April 23, 2020^10^ Italy	59	M	Ischemic heart disease (myocardial revascularization)	Yes/Yes	Yes	Lopinavir/ritonavir enoxaparin/ hydroxychloroquine
April 23, 2020^7^ United States	67	F	Nonischemic cardiomyopathy (LVEF 40%)	Yes/Yes	Yes	Colchicine/hydroxychloroquine/corticosteroid
May 12, 2020^11^ Saudi Arabia	41	F	No history	Yes/Yes	Yes	Azithromycin/ hydroxychloroquine
May 06, 2020^12^ United States	70	F	Ischemic heart disease, hypertension, diabetes	Yes/Yes	Yes	Colchicine

Another case of cardiac tamponade occurred in a 67-year-old female patient with a history of nonischemic cardiomyopathy and left ventricular ejection fraction (LVEF) of 40%; after 1 week of respiratory symptoms, the patient presented with fatigue, hypotension, tachycardia, and low voltage on the electrocardiogram, in addition to normal serum levels of troponin and B-type natriuretic peptide (BNP) markers. Since the symptoms were persistent, an echocardiogram was performed and indicated cardiac tamponade; drainage removed 800 mL of serosanguineous fluid with exudative characteristics. Soon after pericardiocentesis, the clinical, laboratory, electrocardiographic, and echocardiographic findings indicated stress-induced cardiomyopathy characterized by transient apical ballooning (Takotsubo cardiomyopathy).^7^

A 53-year-old female patient, with no previous history of cardiovascular disease, presented with COVID-19 symptoms a week before hospital admission with hypotension and an electrocardiogram showing diffuse ST elevation, in addition to elevated troponin and NT-pro BNP biomarkers, despite angiographically normal coronary arteries. A reverse transcriptase-polymerase chain reaction (RT-PCR) nasopharyngeal swab test was positive for SARS-CoV-2. Cardiac magnetic resonance imaging indicated myocardial edema and late enhancement, as well as circumferential pericardial effusion compatible with myopericarditis.^9^

A 59-year-old male patient admitted with acute coronary syndrome was subjected to a cardiac catheterization that showed multivessel disease and referred to myocardial revascularization. Twenty-two days later, the patient had fever, dyspnea, and a positive RT-PCR COVID-19 test. Chest computed tomography imaging suggested COVID-19-related pneumonia.^10^ On day 23 after infection, the patient developed precordial pain, dyspnea, hypotension, and tachycardia. An echocardiogram showed circumferential pericardial effusion with signs of tamponade in the right heart sections; 250 mL of serosanguineous fluid was drained.

Moreover, a 70-year-old patient with a history of acute coronary artery disease (non-ST elevation myocardial infarction treated 2 weeks before with angioplasty) was admitted with respiratory symptoms. Two days later, the patient had chest pain suggesting acute pericarditis with subsequent cardiac tamponade, requiring intubation and vasoactive drugs due to hemodynamic instability. Hemodynamic and clinical improvement were only achieved after pericardiocentesis.^11^

Another case of COVID-19 and cardiac tamponade involved a 41-year-old patient, with no previous heart diseases, who had sought medical care with respiratory symptoms 10 days prior to hospital admission. The patient showed signs of cardiac tamponade and pericardiocentesis revealed exudative pericardial fluid, with high levels of lactate dehydrogenase and albumin.^12^

Considering these first 6 cases, it is clear that patients with COVID-19 can display pericardial impairment throughout the disease progression. The period of diagnosis and clinical worsening, generally after the first week of infection, matches an increase in inflammatory cytokines, probably with autoimmune mechanisms involved in the etiopathogenesis of pericardial effusion. (O autor pode conferir se esta inclusão está de acordo com o sentido original?)

Some authors believe that, in patients with COVID-19-related myocarditis, the viral infection could be the initial factor that unlocks immune-mediated injury. Necropsy studies detected mononuclear inflammatory cell infiltrates in cardiac tissues, although with no viral inclusion in some cases.^13^ The rapid recovery of cardiac function and only slight increase in viral load suggest that, in addition to viral replication in the myocardium, it is possible that an immune response (including a cytokine storm) could play a significant role in the pathophysiology of this injury.^14^

An excessive release of cytokines is observed in various systemic diseases, including infectious and rheumatic diseases or neoplasms, which can lead to pericardial impairment.^15^

Immunologists and pathologists observed macrophage infiltration in necropsy studies, which may indicate that viremia and the innate immunity control the clinical presentation before lymphocytic infiltration takes place. Inflammatory markers such as C-reactive protein and pro-inflammatory cytokines (IL-6, TNFα, IL8I) are increased.^15^

Therefore, a hyperinflammatory state induced by cytokines can lead to multiple organ failure and could be responsible for the myocardial and pericardial impairment observed in this disease; however, it cannot be disregarded that part of this impairment could also be caused by the viral infection.^16^

In the patient in the Italian case report, a longer period was observed before the onset of pericarditis, and the chemical and cytological analyses of pericardial fluid indicated mostly lymphocytic inflammatory infiltration and the presence of SARS-CoV-2, suggesting a possible direct effect of the virus on cardiac injury. Notably, this patient produced negative RT-PCR blood and nasopharyngeal COVID-19 tests, whereas his pericardial fluid tested positive; this could suggest a possible viral reservoir in the pericardium.^10^ (O estudo não faz menção da nacionalidade do paciente, então sugerimos apenas dizer que o estudo é italiano.)

Five of these cases had cardiac tamponade, which is a high-risk but potentially reversible cause of clinical decompensation. The judgment of whether to adopt an invasive treatment strategy is influenced by the patient’s clinical presentation: in case of arterial hypotension, dyspnea, and paradoxical pulse, the decision is clear. The management of pericardial effusion should consider clinical characteristics (worsening of symptoms), hemodynamics (occurrence or not of tamponade), and an etiological search (infection, tuberculosis, or neoplasm).^5^

## Pharmacological Treatment

The choice of the best pharmacological treatment for pericardial affections in patients with COVID-19 merits further discussion. Nonsteroidal anti-inflammatory drugs (NSAIDs) are the recommended first choices in all acute and recurring cases of pericarditis with no contraindications. Indications include acetylsalicylic acid (ASA) at a dosage of 800 mg every 8 hours or an association of ibuprofen (600 mg every 8 hours) and colchicine.^5^

A retrospective study performed in France^17^ indicated that patients on NSAIDs for symptom control before hospitalization for pneumonia developed more severe forms of the disease and had longer hospitalization periods. Additionally, ibuprofen and acetaminophen were associated with a higher risk of complications in children, especially in high cumulative dosages. The relief of symptoms such as pain and fever by these drugs could be leading to a delay in the introduction of antibiotics.^18^ Patients with more severe viral infections, such as those caused by influenza and SARS-CoV-2, are more likely to use NSAIDs, and the use of ibuprofen could complicate the detection of higher-risk cases.

Controversy regarding the safety of ibuprofen in patients with COVID-19 originated in France, when an infectious disease specialist reported worsening of symptoms in 4 patients that had received this drug; this result was soon supported by the French Health Ministry.^19,20^ Moreover, although it is known that mechanistic evidences are not always confirmed by clinical studies, published data indicated that ibuprofen increased the expression of ACE2 receptors.^20^Altogether, recent epidemiological evidences do not allow the establishment of a causal link for the negative effect of ibuprofen in patients with COVID-19, and current guidelines by the World Health Organization and the American Food and Drug Administration (FDA) do not recommend interrupting the use of ibuprofen in symptomatic COVID-19 cases.^21^ Therefore, the use of this drug should be carefully considered regarding the risks and benefits for symptomatic patients.^22^

Recently, colchicine has been increasingly used along with NSAIDs in treatment strategies for acute and recurring pericarditis, with excellent results; thus, it should also be considered in cases of pericarditis in patients with COVID-19. Its anti-inflammatory effects are related to the disruption of microtubule function, which results in the inhibition of neutrophils and cell adhesion molecules that act in different phases of inflammation.^5^ Regarding its use in patients with COVID-19, at least 8 studies are registered on clinicaltrials.gov for the evaluation of its effects in alleviating systemic and/or myocardial inflammation. Two studies are already in progress: “Colchicine Coronavirus SARS-CoV2 Trial (COLCORONA)” and “The ECLA PHRI COLCOVID Trial. Effects of Colchicine on Moderate/High-risk Hospitalized COVID-19 Patients.”

Corticosteroids are widely used in treatments for acute and recurrent pericarditis since these drugs improve symptoms and reduce inflammatory markers. However, their use is limited to cases of intolerance, contraindications, or failure of treatment with NSAIDs and colchicine due to an increased risk of recurrence, as illustrated by the COPE study.^5^ Corticosteroids are also indicated in specific etiologies such as autoimmune diseases, although in the minimal possible dosages. In these situations, dosage should be slowly reduced only when symptoms are controlled and C-reactive protein levels are normalized. In cases of pericarditis, dosages of 0.25 to 0.5 mg/kg/day should be used.^5^

Usually, the use of corticosteroids is avoided in cases of pericardial affections, whereas NSAIDs and colchicine are recommended. However, COVID-19 has been shown to have a unique pathophysiology, unlocking a state of hyperinflammation and cytokine storm. Considering this situation, the use of corticosteroids could be regarded as the first-choice treatment for pericardial affections in patients with COVID-19, since drugs that reduce inflammation before the impairment of multiple systems have been considered for this disease. Nevertheless, the following question would remain: what is the ideal dosage and period for corticosteroid treatment?^16^

International guidelines for managing and treating sepsis and septic shock indicate that corticosteroids should be used in small doses and short periods of time, and only in cases where fluid replacement and the use of vasopressor drugs were not able to stabilize the patient.^23^ Chen et al.^24^ reported that 19% of patients with pneumonia received methylprednisolone (1-2 mg/kg/day) and dexamethasone for 3 to 15 days (median 5 days). Potential risks of treatment with corticosteroids include delays in viral clearance and the risk of secondary infections.^15^

SARS-CoV-2 was first identified in bronchoalveolar lavage fluid collected from a patient with pneumonia in December 2019, and since then it has been isolated from the respiratory tract, feces, conjunctiva, and blood. More recently, the virus was detected in the pericardial fluid of a patient with cardiac tamponade subjected to pericardiocentesis,^10^ thus the use of corticosteroids would be speculative in this situation.

Some patients with both pericardial affections and COVID-19 were treated with hydroxychloroquine as part of the pericarditis treatment. However, there is no evidence on the efficacy and safety of this drug in these cases. Patients with COVID-19 present systemic inflammation and many develop concurrent myocarditis, possibly leading to ventricular arrhythmias; the use of hydroxychloroquine, known for increasing QT interval, could increase the occurrence of torsade de pointes.

Tocilizumab, an anti-IL-6 receptor monoclonal antibody, is being evaluated in multicentric randomized studies of patients with COVID-19 that present increased IL-6 levels and could benefit from the treatment in case of a cytokine storm, thus reducing myocardial inflammation.

Another noteworthy characteristic of patients with severe forms of COVID-19 is the occurrence of endothelial damage and microvascular thrombosis, which can resemble vasculitis and ultimately lead to the need for amputation. Pathological studies indicated infiltration of monocytes and lymphocytes around vessels presenting hyperplasia and wall thickening, as well as thrombi in microvessels; high levels of antiphospholipid, anticardiolipin, and anti-β2-glycoprotein antibodies were associated with thrombosis.^15^ Therefore, some studies recommend the use of anticoagulant drugs^25^ to avoid thromboembolic events. As previously stated, physicians should be aware of different aspects of each treatment and, in this case, of an increased risk of hemopericardium and tamponade.

An international registry of patients with suspected and confirmed cases of COVID-19 from 69 countries and 6 continents included 1216 cases referred to echocardiography; most of them showed non-specific abnormalities of ventricular dysfunction. Myocardial infarction, myocarditis, and Takotsubo cardiomyopathy were observed only in a few cases. Eleven (1%) of these cases had cardiac tamponade, demonstrating the importance of this issue and thus of complementary examinations such as echocardiography to identify and guide the clinical management and treatment of these patients.^26^

Cardiac surveillance in patients with pericardial impairment is of utmost importance to evaluate recurrences and/or the long-term progression to constrictive pericarditis.
